# Supplementary material for: Time-Course and Tissue-Specific Molecular Responses to Acute Thermal Stress in Japanese Mantis Shrimp Oratosquilla oratoria
Source: Int J Mol Sci. 2023 Jul 26;24(15):11936. doi: 10.3390/ijms241511936 (PMC10419158; doi:10.3390/ijms241511936)
Supplement: Supplementary file 1 [file ijms-24-11936-s001.zip › ijms-2460856-Supplementary.pdf]

Supplemental information for

Time-Course and Tissue-Specific Molecular Responses to Acute Thermal Stress in Japanese Mantis Shrimp *Oratosquilla oratoria*

Liwen Zhang [1,2,3,4], Zhongli Sha [1,2,3,4,*] and Jiao Cheng [1,2,*]

[1] Laboratory of Marine Organism Taxonomy and Phylogeny, Qingdao Key Laboratory of Marine Biodiversity and Conservation, Institute of Oceanology, Chinese Academy of Sciences, Qingdao 266071, China; zhangliwen@qdio.ac.cn

[2] Laboratory for Marine Biology and Biotechnology, Qingdao National Laboratory for Marine Science and Technology, Qingdao 266237, China

[3] Shandong Province Key Laboratory of Experimental Marine Biology, Institute of Oceanology, Chinese Academy of Sciences, Qingdao 266071, China

[4] University of Chinese Academy of Sciences, Beijing 100049, China

* Correspondence: shazl@qdio.ac.cn (Z.S.); jcheng@qdio.ac.cn (J.C.)

Supplementary data

Table S1. Transcriptome sequencing data of *Oratosquilla oratoria*.

Table S2. List of functional differentially expressed transcripts in (A) gill, (B) hepatopancreas, and (C) muscle.

Table S3. GO enrichment results of transcripts in five hub modules. The important GO terms related to acute thermal response are in bold black.

Table S4. List of differentially expressed transcripts associated with translation and metabolism.

Table S5. PCR primers for the validation of RNA-Seq data by qRT-PCR.

Figure S1. The 12h LT50 test under heat stress of *O. oratoria*. The columns and line charts represent the survival number and survival rates of *O. oratoria* at different heat stress temperatures. The red arrow points to the 12h LT50 of *O. oratoria*.

Figure S2. Transcript expression pattern in muscle, gill, and hepatopancreas of *O. oratoria* at different heat stress time points revealed by (A) Principal components analysis and (B) Pearson correlation analysis. The correlation is represented by color; darkred represents a stronger correlation. (C) The heatmaps above arrows represent the expression patterns of all transcripts throughout the whole heat stress process in muscle, gill, and hepatopancreas, respectively; while the heatmaps below arrows represent the expression patterns of DETs identified in the muscle, gill, and hepatopancreas transcriptomes throughout the heat stress process, respectively.

Figure S3. The Venn diagram of differentially expressed transcripts at three time points in muscle, hepatopancreas, and gill of *O. oratoria* (A), and in three tissues at 0.5h, 6h, and 12h (B), respectively. Numbers in red, blue, and black denote up-, down- and anti-directionally regulated transcripts, respectively.

Figure S4. Heatmap results of Kyoto Encyclopedia of Genes and Genomes (KEGG) enrichment analysis of (A) up- and (B) down-regulated DETs in three tissues at three heat-shock timepoints.

Figure S5 Comparison of transcript expression levels obtained by RNA-Seq and qRT-PCR.

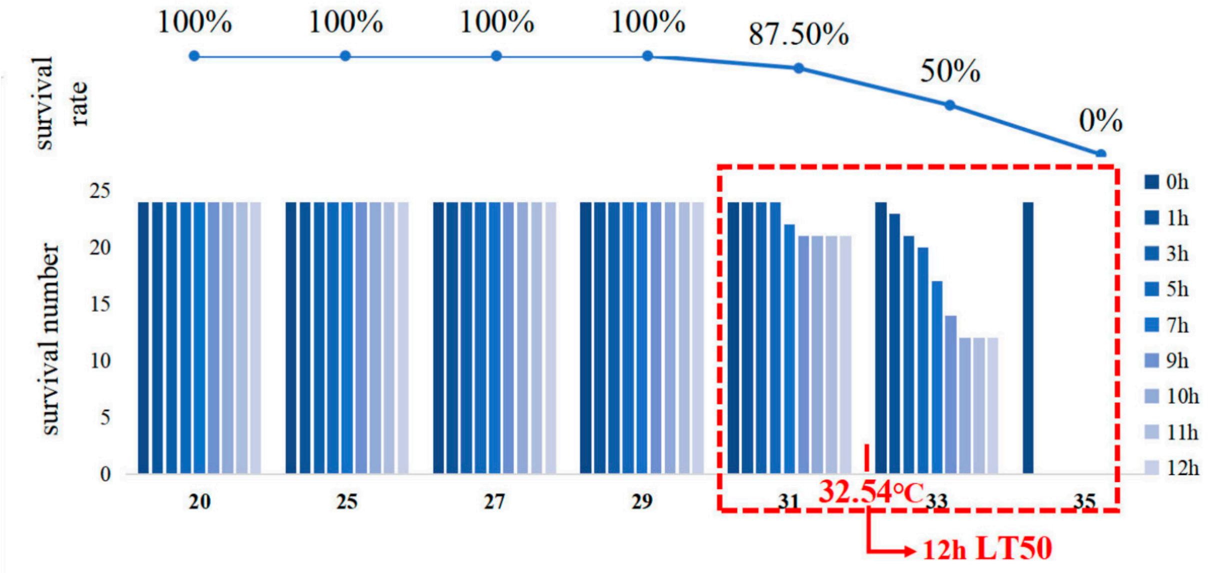

Figure S1. The 12h LT50 test under heat stress of *O. oratoria*. The columns and line charts represent the survival number and survival rates of *O. oratoria* at different heat stress temperatures. The red arrow points to the 12h LT50 of *O. oratoria*.

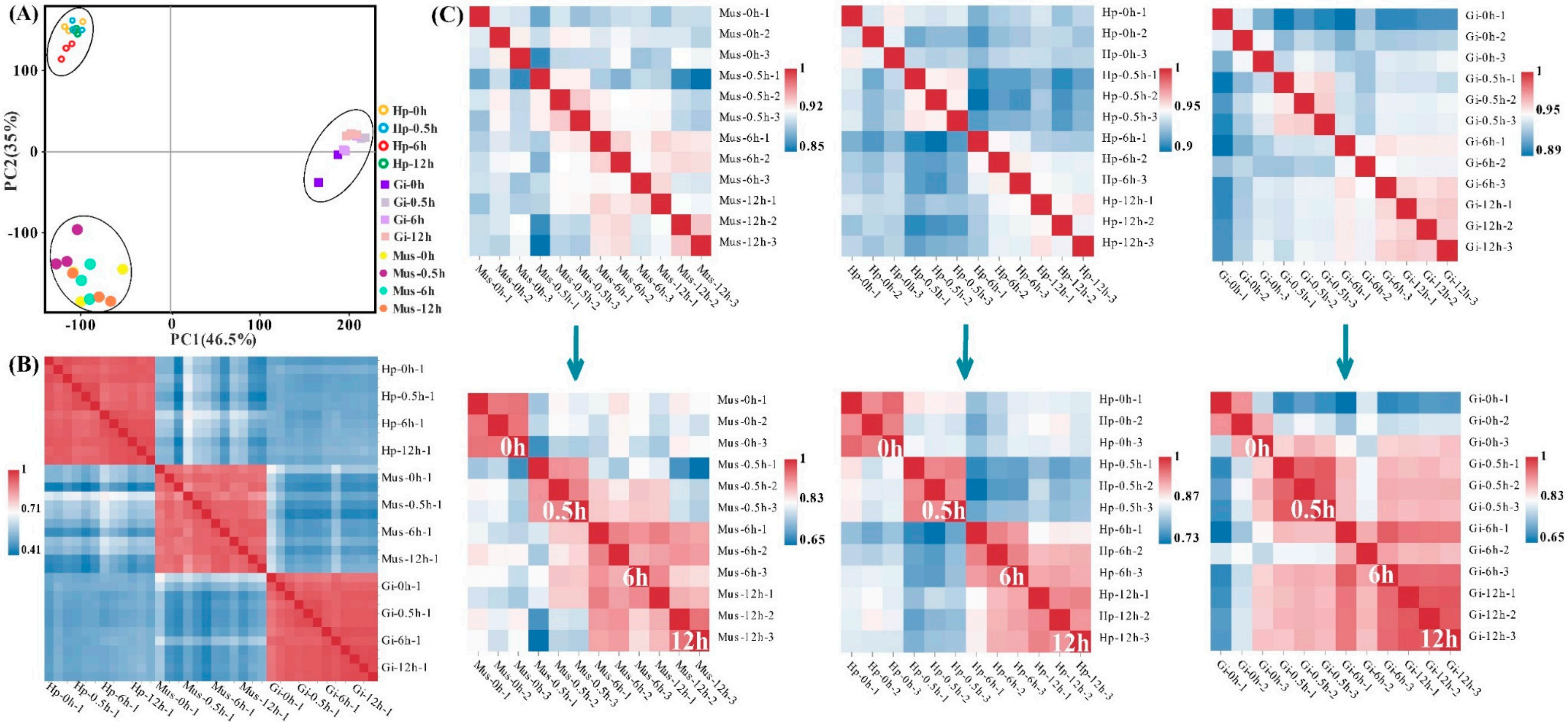

Figure S2 Transcript expression pattern in muscle, gill, and hepatopancreas of *O. oratoria* at different heat stress time points revealed by (A) Principal components analysis and (B) Pearson correlation analysis. The correlation is represented by color; darkred represents a stronger correlation. (C) The heatmaps above arrows represent the expression patterns of all transcripts throughout the whole heat stress process in muscle, gill, and hepatopancreas; while the heatmaps below arrows represent the expression patterns of muscle's, gill' s and hepatopancreas' DETs throughout the heat stress process, respectively.

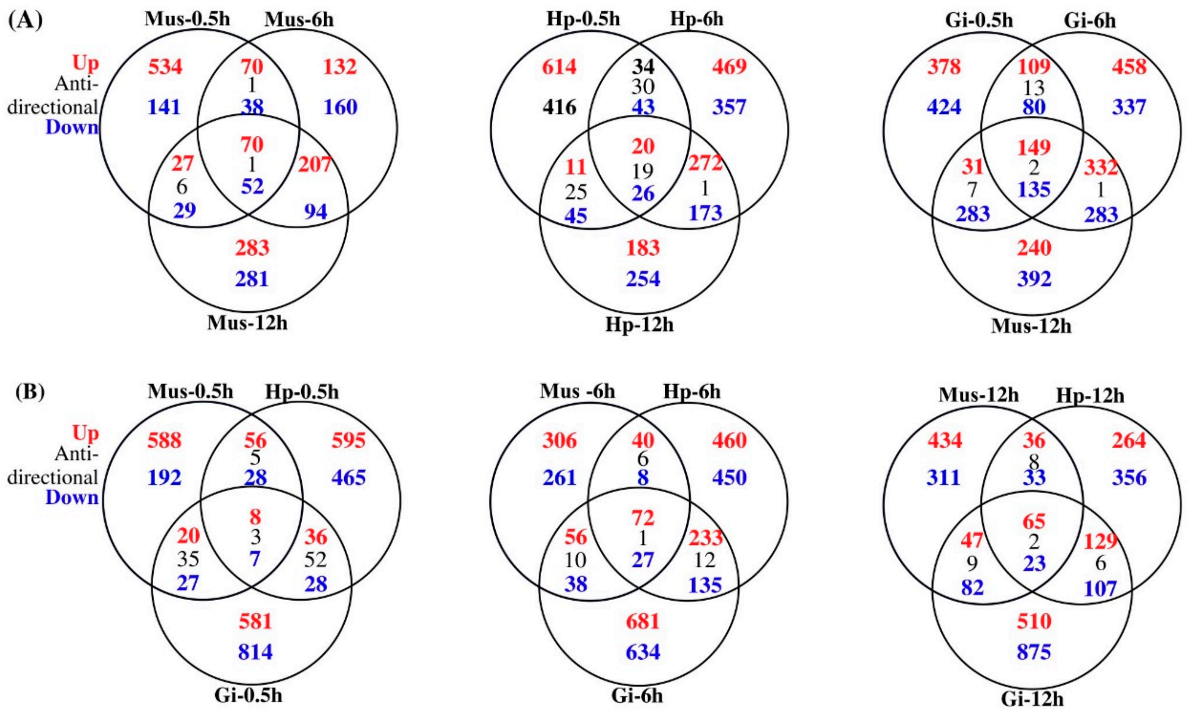

Figure S3. The Venn diagram of DETs (A) at three time points in muscle, hepatopancreas, and gill of *O. oratoria*, respectively, and (B) in three tissues at 0.5h, 6h, and 12h, respectively. Numbers in red, blue, and black denote up-, down- and anti-directionally regulated transcripts, respectively.

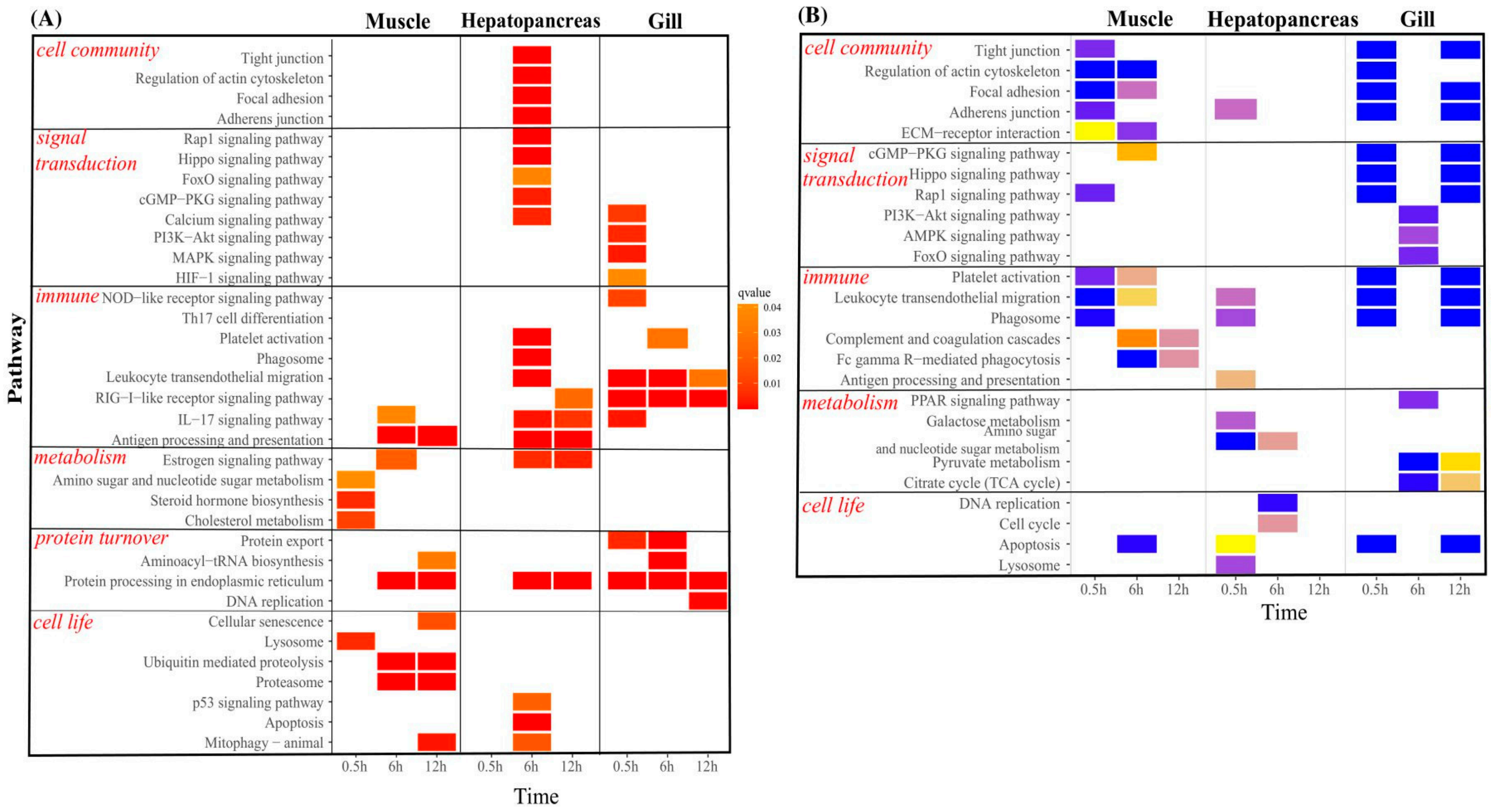

Figure S4. Heatmap results of Kyoto Encyclopedia of Genes and Genomes (KEGG) enrichment analysis of (A) up- and (B) down-regulated DETs in three tissues at three heat stress time points.

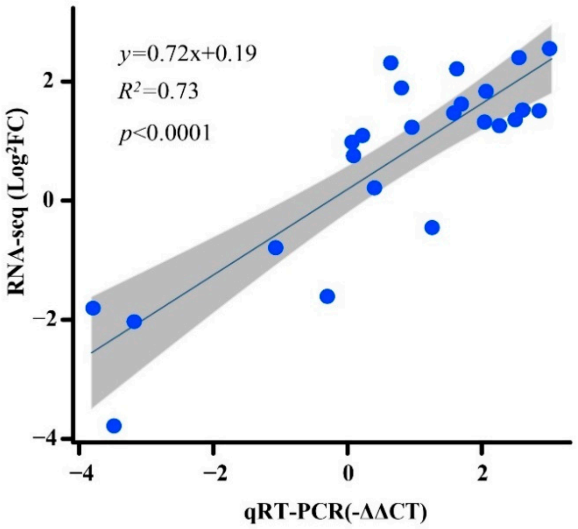

Figure S5. Comparison of gene expression levels obtained by RNA-seq and qRT-PCR. $\log_2$FC values were calculated for 8 DETs and a relatively high correlation ($R^2 > 0.73$) was observed between the results obtained using the two techniques.
